# Investigating the Effects of Metabolic and Bariatric Surgery on Systemic Immune‐Inflammation Index and Its Relationship With Smoking

**DOI:** 10.1002/wjs.12499

**Published:** 2025-02-06

**Authors:** Hatice Toprak, Şükrü S. Toprak

**Affiliations:** ^1^ Faculty of Medicine Department of Anesthesiology and Reanimation Karamanoğlu Mehmetbey University Karaman Türkiye; ^2^ Faculty of Medicine Department of General Surgery Karamanoğlu Mehmetbey University Karaman Türkiye

**Keywords:** bariatric surgery, inflammation, obesity, systemic immune‐inflammation index

## Abstract

**Purpose:**

The contribution of obesity to inflammation may play a role in the progression of obesity‐associated medical problems. The systemic immune inflammation index (SII) has recently been identified as a prognostic indicator for many adverse conditions. The primary purpose of the present study was to investigate the effects of metabolic and bariatric surgeries on white blood cell (WBC), platelet (PLT), lymphocyte (LYN), neutrophil (NEU), neutrophil/lymphocyte (NLR), platelet/neutrophil (PLR), and systemic immune inflammation index (SII). The secondary aim was to evaluate the effects of sleeve gastrectomy (SG) and gastric bypass (GB) surgeries, the most commonly performed metabolic and bariatric procedures, on individual inflammation parameters and their relationship with smoking status.

**Methods:**

The blood inflammatory markers of the participants who underwent surgery were analyzed using the data evaluated during routine clinic follow‐ups in the preoperative period and postoperative 1st, 3rd, 6th, and 12th months.

**Results:**

The primary result was a statistically significant decrease in WBC, NEU, NLR, and SII values in the 3rd postoperative month in those who underwent metabolic and bariatric surgery (MBS) (***p*** values for each parameter: 0.000, 0.000, 0.028, and 0.006, respectively). A statistically significant decrease in WBC, NEU, and SII values in the 3rd postoperative month compared to preoperative values in nonsmoking individuals with obesity who underwent sleeve gastrectomy surgery was presented as our secondary result (***p*** values for each parameter: 0.000, 0.000, and 0.015, respectively).

**Conclusion:**

In our study, MBS provided significant regression in inflammation parameters at 3 months after surgery in people smoking less than 10 cigarettes per day, although this effect did not seem to persist long term.

**Clinical Trial Registration:**

ACTRN12623000162617

## Graphical abstract

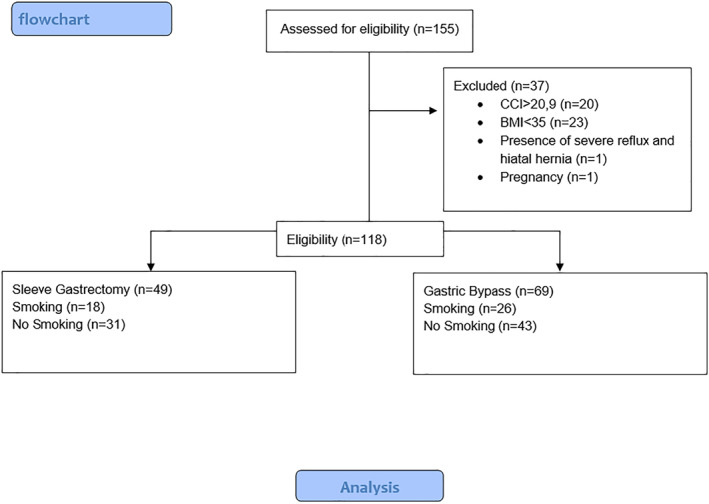

## 1 Introduction

Obesity is associated with medical problems such as hypertension, diabetes, and obstructive sleep apnea [1]. Obesity‐related inflammation was first described by Hotamışlıgil et al. in 1993 [2]. Individuals who are obese tend to maintain a state of chronic low‐grade inflammation [3]. It is already known that obesity‐related chronic inflammation contributes to the progression of chronic diseases [4]. Studies are encouraged to develop strategies to prevent the emergence and progression of obesity‐related diseases, which can be defined as metabolic‐driven immunological diseases that have complex pathophysiologies [5].

White blood cell count (WBC), neutrophil count (NEU), neutrophil–lymphocyte ratio (NLR), and platelet–lymphocyte ratio (PLR) obtained from routine blood tests for inflammation were calculated previously and used as prognostic indicators [6, 7, 8]. Markers that contain one or two types of immune‐inflammatory cells may not reflect the inflammatory state adequately. The systemic immune‐inflammation index (SII), which provides novel data for inflammation, was developed by Hu et al. in 2014 [9]. Elevated SII values were presented as a poor prognosis indicator for many clinical conditions [9, 10, 11, 12]. It has been argued that SII, which combines independent white blood cells and platelets, reflects the thrombocytosis‐inflammation‐immunity interaction [11].

Metabolic and bariatric surgery (MBS) provided long‐term effectiveness in weight loss and yielded satisfactory results in the remission of comorbid diseases that are associated with cardiovascular risk and obesity [1, 13, 14]. Patients with obesity have a higher risk of developing OSA [15]. OSA may increase oxidative stress in endothelial cells with intermittent hypoxia and exacerbate inflammation [16, 17]. External factors (e.g., smoking) contribute to the development of obesity‐related diseases and inflammation [18]. MBS can promote the significant conversion of immune cells from a pro‐inflammatory state into an anti‐inflammatory one in the adipose tissue [19]. The purpose of the present study was to investigate the effects of MBS on inflammatory indicators obtained from routine blood tests and its relationship with smoking. According to the hypothesis of the study, MBS will make positive contributions to inflammatory indicators, and smoking will reduce this effect.

## 2 Materials and Methods

**FIGURE 1:**
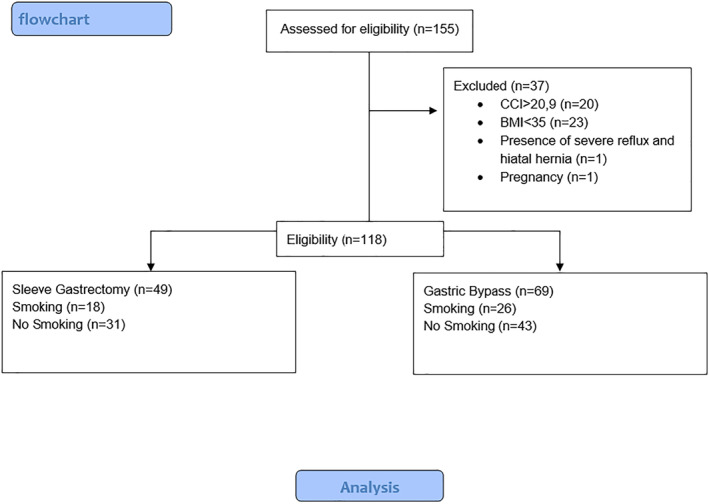
The flowchart. CCI: comprehensive complication index and BMI: body mass index.

The study had a single‐center, retrospective, observational design and was evaluated at the Karamanoğlu Mehmetbey University Faculty of Medicine Ethics Committee. Ethical approval was received with the decision 10‐2022/08. It was registered to ANZCTR with the number ACTRN12623000162617. Permission was received from the Karaman Training and Research Hospital Board to conduct the study in our hospital. The Consolidated Standards of Reporting Trials (CONSORT) checklist was used (Figure 1).

The data obtained from the laboratory records of the preoperative and postoperative periods of patients who underwent MBS in our hospital between May 2021 and September 2023 were evaluated.

The Clavien–Dindo classification system (CDC) was used in the evaluation of the complications [20]. The comprehensive complication index (CCI) is based on the CDC calculator and is a tool that supports the assessment of the overall morbidity of patients in the postoperative period. The morbidity rate is reflected on a scale from “0” (no complications) to “100” (mortality). CCI scores were provided by an online calculator [21]. It can be thought that experiencing complications related to CCI > 20.9 may affect inflammation parameters.

### 2.1 Inclusion Criteria

The individuals who underwent sleeve gastrectomy (SG) or gastric bypass (GB) surgery between the ages of 18 and 65 were included in the study. The patients who were informed that their surgery‐related data could be used for scientific purposes in the preoperative period and whose consent was obtained were evaluated in the study.

### 2.2 Exclusion Criteria

Patients who could not read or write Turkish, those who had psychiatric disorders, those who did not allow the use of their data for scientific purposes, those who had undergone surgical techniques other than laparoscopy, those who had had revision bariatric surgeries with BMI < 35 kg/m^2^, the presence of severe reflux and hiatal hernia [14], patients with a CCI value of 20.9% and above in the CCI calculator recorded in the Clavien–Dindo calculator complication grading system, and patients who experienced pregnancy in the postoperative period were excluded from the study.

Gastroesophageal reflux disease (GERD) and hiatal hernia were associated with local and systemic inflammation in many previous studies [14, 22]. They might contribute to systemic inflammation by releasing various inflammatory mediators [22]. Proinflammatory factors (e.g., interleukin‐6 and ‐8, leukocytes, and oxidative stress) were also shown to play roles in the development of gastroesophageal reflux disease (GERD) [23]. Considering this complex relationship, GERD and hiatal hernia patients were not included in the study.

SG and GB surgeries were performed laparoscopically by a single surgeon. A large part of the stomach was resected vertically in SG, and a small pouch of the stomach was connected to the small intestine, bypassing the proximal part of the duodenum and jejunum in GB [24]. Postoperative follow‐up of the patients was performed by the surgeon who performed the surgery under the same conditions for all patients. Complications of the patients were determined during bedside visits and supported by the hospital’s electronic database in the first postoperative month.

### 2.3 Data Collection

The age, sex, smoking status, preoperative comorbidities (diabetes, hypertension, asthma, etc.), and the presence of obstructive sleep apnea (OSA) of the patients were questioned. The patients whose presence of OSA was confirmed by the previous diagnosis were evaluated as “OSA (positive).” OSA (positive) was determined by patient declaration during preoperative evaluation. Those who had smoked 10 or more cigarettes per day for at least 1 year before the surgery were recorded as having a smoking status.

The data were accessed from the electronic database of our hospital. Complication grading of patients with MBS evaluated at the end of the postoperative 1st month was achieved by screening the data registered in the online CDC system.

### 2.4 Exposure Variable

Preoperative data and postoperative first‐, third‐, sixth‐, and twelfth‐month data of patients with MBS were obtained by scanning the electronic database of our hospital and the data registered in the CDC online system. The data with a ± 10‐day difference during follow‐ups were included. Lymphocyte (LYN), neutrophil (NEU), and platelet (PLT) counts (expressed as × 10^3^ cells/μL) measured with automatic hematology analyzers were recorded. Systemic immune‐inflammation index (SII) was calculated by using two different white blood cell and PLT counts.

The following formula was utilized to calculate SII:

SII = (PLT count × NEU count)/LYN count [9].

### 2.5 Primary Outcome

The primary aim of this study was to investigate the effects of MBS on inflammatory markers, WBC, PLT, LYN, NEU, NLR, PLR, and SII, at postoperative months 1, 3, 6, and 12.

### 2.6 Secondary Outcome

The secondary target of the present study was to investigate the effects of SG and GB surgeries separately on inflammation parameters and their relationship with smoking status.

### 2.7 Statistical Analysis

The mean and standard deviation values were given as descriptive statistics for numerical variables, and frequency and percentage values were given as descriptive statistics for categorical variables. The chi‐square test was used to analyze categorical variables. The *t*‐test and mixed effects models were used in the analysis of numerical variables. Post‐hoc comparisons were made by using the least square means (with Tukey correction when necessary). The data analysis was performed with the *R* 4.3.2 program, and ***p*** < 0.05 was considered significant.

## 3 Results

A total of 155 patients were evaluated. The flowchart of the study is given in Figure 1. Basic demographic and clinical data of the participants are given in Table 1.

**TABLE 1:** Demographic data and clinical characteristics.

Variable	*N* = 118^a^
Age	38.86 ± 10.42
Comorbidity	
Yes	48.00 (40.68%)
None	70.00 (59.32%)
Sex	
Male	33.00 (27.97%)
Female	85.00 (72.03%)
Smoking status	
Yes	44.00 (37.29%)
None	74.00 (62.71%)
Body length (cm)	164.80 ± 9.26
Body weight (kg)	122.97 ± 19.70
OSA	
Yes	20.00 (16.95%)
None	98.00 (83.05%)
Operation type	
Sleeve gastrectomy	49.00 (41.53%)
Gastric bypass	69.00 (58.47%)
BMI (kg/m2)	45.19 ± 5.69

Abbreviations: BMI, Body Mass Index (kg/m2); OSA, Obstructive Sleep Apnea.

^a^ Mean ± SD; *n* (%).

The relationship between the demographic data and clinical characteristics of the patients and their smoking status is given in Table 2.

**TABLE 2:** Comparison of patients' different demographic characteristics and smoking status.

Variable	Smoking status *N* = 44^a^	None smoking status *N* = 74^a^	*p* ^b^
Age	37.57 ± 8.37	39.62 ± 11.45	0.3
Comorbidity			0.008*
Yes	11.00 (25.00%)	37.00 (50.00%)	
None	33.00 (75.00%)	37.00 (50.00%)	
Sex			0.005*
Male	19.00 (43.18%)	14.00 (18.92%)	
Female	25.00 (56.82%)	60.00 (81.08%)	
Body length (cm)	167.36 ± 8.61	163.27 ± 9.35	0.017*
Body weight (kg)	126.16 ± 19.71	121.07 ± 19.58	0.2
OSA			0.2
Yes	10.00 (22.73%)	10.00 (13.51%)	
None	34.00 (77.27%)	64.00 (86.49%)	
Operation type			> 0.9
Sleeve gastrectomy	18.00 (40.91%)	31.00 (41.89%)	
Gastric bypass	26.00 (59.09%)	43.00 (58.11%)	
BMI (kg/m^2^)	44.92 ± 5.27	45.36 ± 5.96	0.7

*Note:* *p‐value is below the threshold of < 0.05.

Abbreviations: BMI: Body Mass Index (kg/m2); OSA, Obstructive Sleep Apnea.

^a^ Mean ± SD; *n* (%).

^b^ Welch Two Sample *t*‐test; Pearson's Chi‐Square Test.

**FIGURE 2:**
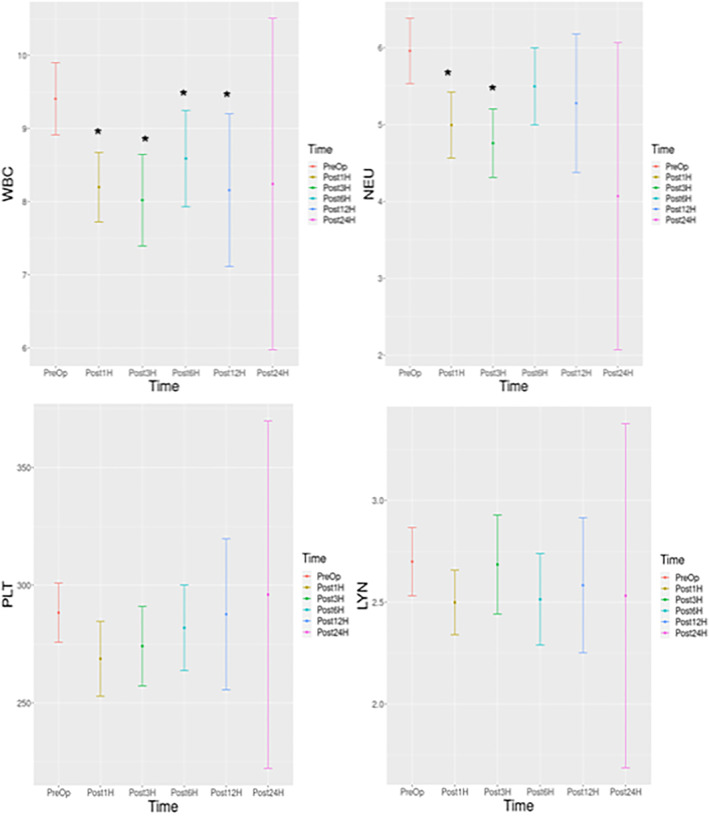
Histogram of change over time for WBC, PLT, LYN, and NEU. WBC: white blood cell, PLT: platelet, LYN: lymphocyte, NEU: neutrophil, Preop: preoperative, Post1: postoperative 1st month, Post3: postoperative 3rd month, Post1: postoperative 6th month, Post12: postoperative 12th month, and Post24: postoperative 24th month. *p‐*value is below the threshold of < 0.05.

**FIGURE 3:**
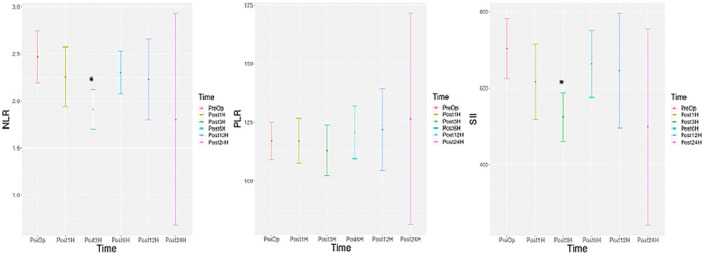
Histogram of change over time for NLR, PLR, and SII. NLR: neutrophil/lymphocyte, PLR: platelet/neutrophil, SII: (platelet count × neutrophils count)/lymphocyte count, Preop: preoperative, Post1: postoperative 1st month, Post3: postoperative 3rd month, Post1: postoperative 6th month, Post12: postoperative 12th month, and Post24: postoperative 24th month. * *p‐*value is below the threshold of < 0.05.

The changes in the inflammatory indicators over time were examined in all patients who underwent MBS, regardless of the surgery types (Figures 2, 3). A statistically significant decrease was detected for WBC in the postoperative 1st , 3rd , 6th , and 12th months compared to preoperative values (***p*** values: 0.000, 0.000, 0.004, and 0.023, respectively). A statistically significant decrease was detected in NEU in the 1st and 3rd postoperative months compared to the preoperative values (***p*** values: 0.000 and 0.000, respectively). A statistically significant decrease was detected in NLR in the 3rd postoperative month compared to preoperative values (***p*** = 0.028). A statistically significant decrease was detected in SII in the 3rd postoperative month compared to preoperative values (***p*** = 0.006).

**FIGURE 4:**
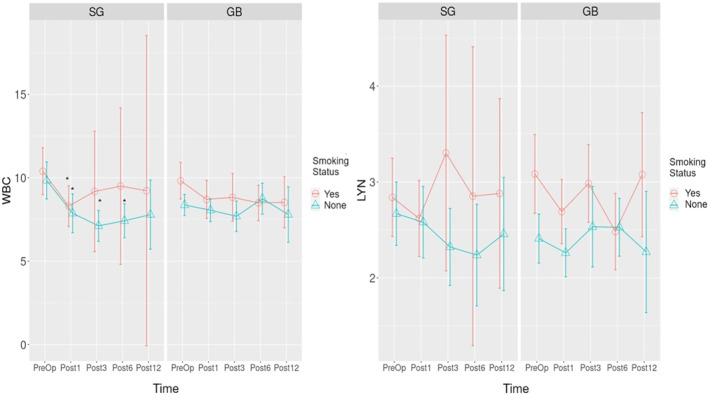
Time‐dependent change graph of WBC and LYN for sleeve gastrectomy and gastric bypass operations. WBC: white blood cell, LYN: lymphocyte, Preop: preoperative, Post1: postoperative 1st month, Post3: postoperative 3rd month, Post1: postoperative 6th month, Post12: postoperative 12th month, and Post24: postoperative 24th month. * *p*‐value is below the threshold of < 0.05.

The time‐dependent change graph for WBC for SG and GB surgeries is given in Figure 4. According to the analysis, a statistically significant decrease was detected in preoperative WBC values in nonsmoking patients who underwent SG surgery in the postoperative 1st , 3rd , and 6th months (***p*** values: 0.003, 0.000, and 0.010, respectively). When the time‐dependent change in WBC was examined in smokers who underwent SG surgery, a significant decrease was detected in the first postoperative month (***p*** = 0.010).

**FIGURE 5:**
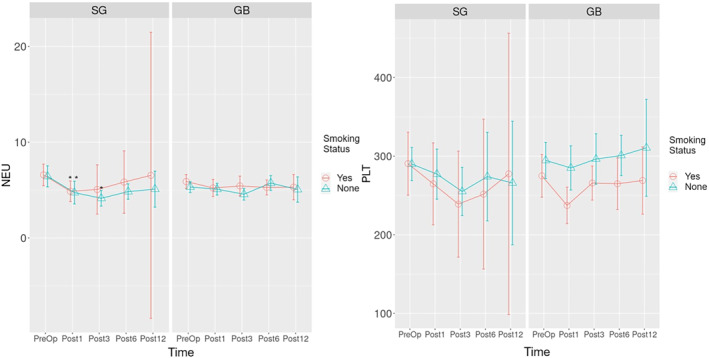
Time‐dependent change graph of NEU and PLT count for sleeve gastrectomy and gastric bypass operations. PLT: platelet, NEU: neutrophil, Preop: preoperative, Post1: postoperative 1st month, Post3: postoperative 3rd month, Post1: postoperative 6th month, Post12: postoperative 12th month, and Post24: postoperative 24th month. * *p*‐value is below the threshold of < 0.05.

The time‐dependent change graph of NEU and PLT for SG and GB surgeries is given in Figure 5. According to the analysis, a statistically significant decrease was detected in the postoperative 1st and 3rd months in nonsmoking patients who underwent SG surgery compared to preoperative NEU values (***p*** values: 0.006 and 0.000, respectively). When the time‐dependent change of NEU in smokers who underwent SG surgery was examined, a significant decrease was detected in the first postoperative month (***p*** = 0.040).

**FIGURE 6:**
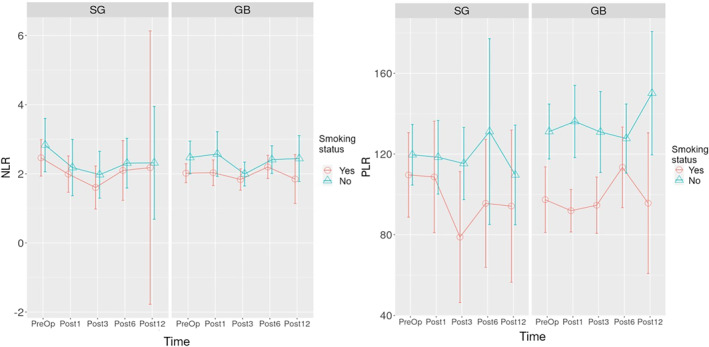
Time‐dependent change graph of NLR and PLR count for sleeve gastrectomy and gastric bypass operations. NLR: neutrophil/lymphocyte, PLR: platelet/neutrophil, Preop: preoperative, Post1: postoperative 1st month, Post3: postoperative 3rd month, Post1: postoperative 6th month, Post12: postoperative 12th month, and Post24: postoperative 24th month. * *p*‐value is below the threshold of < 0.05.

The time‐dependent change graph of NLR and PLR for SG and GB surgeries is given in Figure 6. According to the analysis, the change over time was not significant at any time point in those who underwent SG and GB surgeries.

**FIGURE 7:**
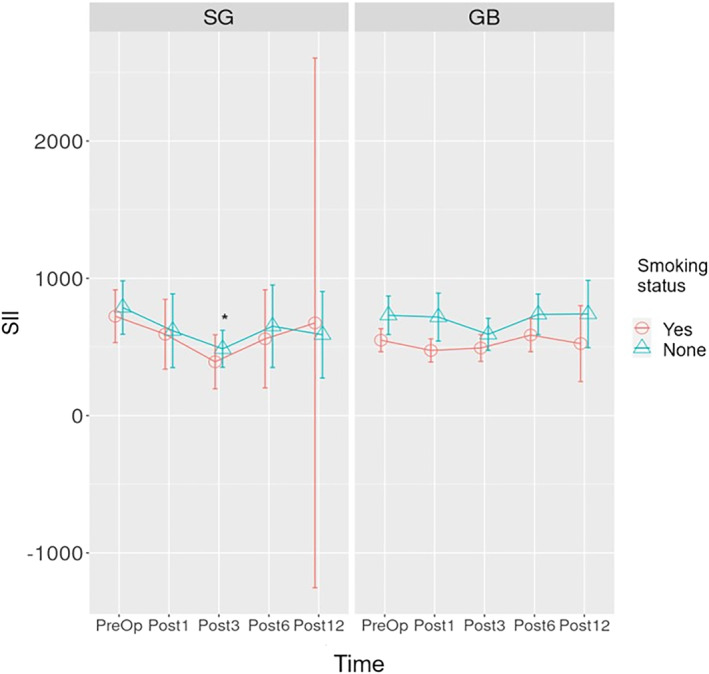
Time‐dependent change graph of SII count for sleeve gastrectomy and gastric bypass operations. SII: (platelet count × neutrophils count)/lymphocyte count, Preop: preoperative, Post1: postoperative 1st month, Post3: postoperative 3rd month, Post1: postoperative 6th month, Post12: postoperative 12th month, and Post24: postoperative 24th month. * *p*‐value is below the threshold of < 0.05.

The time‐dependent change graph of SII for SG and GB surgeries is given in Figure 7. According to the analysis, a statistically significant decrease was detected in the postoperative 3rd month compared to preoperative SII values in nonsmoking patients who underwent SG surgery (***p*** = 0.015).

## 4 Discussion

In this study, the changes in blood markers (WBC, LYN, NEU, PLT), SII, NLR, and PLR [25, 26, 27] values, which are employed as inflammatory markers for many adverse conditions, including complications after bariatric surgery, were examined after MBS. We also evaluated the relationship of this change with our study variables, namely, the difference in operation type and smoking, by performing a subgroup analysis. Our findings were presented as our secondary results, which show that the operation type and smoking status affected the markers. A statistically significant decrease was found in WBC, NEU, and SII values ​​in the 3rd month postoperatively in nonsmokers who underwent sleeve gastrectomy (SG). This difference was not observed in smokers.

The primary purpose of the study was to examine the effects of MBS on WBC, PLT, LYN, NEU, NLR, PLR, and SII, regardless of operation type.

Statistically significant decreases were detected in WBC, NEU, NLR, and SII values in 3 months after MBS compared to the preoperative period (*p* values: 0.000, 0.000, 0.028, and 0.006, respectively).

The secondary purpose of the study was to investigate the effects of sleeve gastrectomy (SG) and gastric bypass (GB) surgeries separately on inflammation parameters and their relationship with smoking status.

After SG operation, statistically significant decreases were detected in the 3^rd^ month in those who did not smoke in WBC, NEU, and SII values compared to the preoperative period (*p* values: 0.000, 0.000, and 0.015, respectively). This difference was not observed in those who smoked.

In this study, the temporal changes in blood inflammatory markers of patients with MBS were analyzed, and a statistically significant decrease was detected in WBC, NEU, NLR, and SII in a short period of 3 months after MBS when compared to preoperative values ((***p*** < 0.05 for each; Figure 2 and Figure 3). This result may reflect the effect of weight loss provided by bariatric surgeries on the markers. We think that the results were consistent with a previous study that evaluated the effects of SG [28]. We think that our NEU‐related results, even without density‐related data, support the previously presented results of Pino et al., who reported a decrease in low‐density neutrophil rates (LDN) in MBS [29].

Statistically significant differences in inflammation markers in bariatric surgeries in a short time (e.g., 1 month) cannot be explained only by weight loss. This effect may be associated with the anatomical and physiological effects of different surgeries. In our study, inflammation parameters and their relationship with smoking for SG and GB surgeries were investigated separately (the secondary outcome) (Figure 4, Figure 5, Figure 6, and Figure 7). Lo et al. found decreased values in WBC in the postoperative 3rd and 12th months in SG surgeries regardless of smoking status [30]. Sen et al. reported a significant decrease in WBC and PLT numbers in the 12th month [31]. Our results can be considered compatible with previous studies regarding WBC. We think that our results show that SG surgery is more effective in reducing the WBC parameter, but this effect decreases with smoking.

The number of NEUs in nonsmoking individuals with obesity who underwent SG surgery was found to be statistically and significantly lower in the 1st and 3rd postoperative months compared to preoperative values in this study (Figure 5). Our results are compatible with the results of the study conducted in patients who had previously undergone LSG surgery [30]. Lo et al. also reported statistically significant decreases in NLR. We found a decrease in NLR in our study, but this effect was not statistically significant (Figure 6). The NEU decrease showed a significant difference in the first postoperative month in smokers with obesity. The decreased SII in the 3rd postoperative month compared to preoperative values, which we think was associated with the decreased number of NEUs, was not found in smoking patients who underwent SG surgery (Figure 7).

The positive contributions of MBS to chronic inflammation were demonstrated in previous studies [28, 29]. SII levels might be used as an easily detectable biomarker of systemic inflammatory activity [32]. There is no sufficient data in the literature regarding the relationship between SII and low‐grade chronic inflammation caused by obesity. Inflammatory responses to sex hormones may be different. Estrogen may exacerbate the inflammatory response in individuals. In general, androgens show anti‐inflammatory effects, whereas estrogens produce pro‐inflammatory effects [33]. Comorbidities such as DM, HT, hyperlipidemia, OSA, and COPD in the participants might contribute to systemic inflammation [3, 34, 35]. The fact that the effect expected to be long lasting in our study was limited to the 3rd month might be associated with the large number of female patients in our sample, the fact that comorbidities were not excluded, and the difference in compliance with the lifestyle changes that change with MBS in individuals with obesity.

Although we did not evaluate it in our study, possible decreases in ghrelin and leptin hormones after MBS might have contributed to these effects. Ghrelin stimulates appetite [36] and is produced in the stomach corpus [37]. It plays a role in regulating the immune response [38]. Leptin is effective in controlling appetite [39] and is released from the adipose tissue [40]. It can also regulate energy balance and modulate inflammation [39]. There is a situation in favor of women for leptin levels [41]. The high number of female participants in our study might have affected inflammation parameters by causing increased leptin levels.

It cannot be excluded that the recovery of OSA after MBS may have been reflected in our results. This possibility may be expected to be effective in the long postoperative period associated with postoperative weight loss. The favorable results shown in the first month of postoperative inflammation parameters in our study suggest that there may be different mechanisms other than the effect of OSA.

E‐cigarette or vaping‐induced lung injury (EVALI) has been a topic of discussion in recent years. In addition, a previous study associated different tobacco use habits with increased cardiovascular risks [42]. The pathophysiology has not been fully explained yet. The possible mechanism is that inhaled chemicals cause cytotoxicity and neutrophilic inflammation [43]. There were no participants with different tobacco use habits in this study.

Postoperative pain might be more severe in patients undergoing open surgery, and analgesia regimens might differ from laparoscopic surgeries for nonsteroidal anti‐inflammatory drugs (NSAIDs). In addition, open surgeries are riskier than laparoscopic surgeries in terms of wound infection. For these reasons, patients who underwent open surgery were not included in our study.

The fact that our study was performed without excluding patients with comorbidities can be considered a limitation. We think that the fact that the comorbidities of the operated individuals were in remission reduced this effect. While planning the study, we considered testing with a control group as one of our goals. However, the sample was not suitable for this. We hope future studies will be conducted with obese individuals who do not have any comorbidity as a control group to contribute to the literature data.

Patients who were using steroids and immunomodulators for various reasons (COPD, rheumatologic diseases, psoriasis, skin diseases, etc.) could not be excluded because of a lack of data in the retrospective screening of patients. This is a limitation of the study. We hope that prospectively designed studies that will eliminate this limitation will be added to the literature in the future.

As far as we are concerned, the lowest category of information regarding the number of cigarettes smoked per day is 1–10 cigarettes. Information on a low number and intermittent smoking is limited [44]. We thought that the low dose‐response relationship in our study might mask our results, and therefore, we investigated the effects of smoking 10 or more cigarettes per day for at least 1 year on inflammatory parameters by referring to previous studies [44, 45].

It is already known that smoking increases the risk of developing postoperative complications [46]. Chow et al. reported that smoking within 1 year before bariatric surgery is associated with postoperative morbidity in bariatric surgeries [47]. During the preoperative anesthesia evaluations, individuals who were scheduled to undergo MBS surgery were informed and advised to quit smoking. Quitting smoking may not be possible for every patient. The preoperative data evaluated in our study were the last data evaluated just before the surgery. In our data, smoking status was questioned as number/day/year. The fact that our patients who quit or reduced their smoking were not evaluated can be considered as our limitation. We believe that future studies without this limitation will be added to the literature.

We consider the relatively high number of patients and the fact that the surgeries and postoperative follow‐ups were standardized and performed by a single surgeon as the strong points of our study. We considered the limitations as the fact that the possibility of individuals with obesity undergoing MBS surgery had received preoperative treatment could not be evaluated, the possibility of comorbidities contributing to the inflammation status despite being in remission was not excluded, their relationship with postoperative weight loss could not be investigated, the number of participants who were female because of the nature of MBS, and the shortness of the follow‐up period.

## 5 Conclusion

The present study aimed to investigate the effects of MBS operation on blood inflammation markers in obese individuals. The difference in operation type and the relationship between smoking status and inflammation were given as secondary results. As a result of the study, a decrease was detected in WBC, NEU, NLO, and SII values ​​in the postoperative 3rd month after MBS. It was shown that these effects changed with the operation type and smoking status. The study provided findings that systemic inflammation caused by obesity would decrease after MBS. These effects were greater in SG operation and smoking status reduced these effects. The importance of questioning smoking status and warnings to quit smoking in preoperative anesthesia evaluations was emphasized. The contributions of age, sex, and comorbidities to inflammation could not be explained in our study. We hope that future studies will eliminate our limitations and investigate these issues in a detailed manner. In our study, MBS provided significant regression in inflammation parameters at 3 months after surgery in people smoking less than 10 cigarettes per day, although this effect did not seem to persist long term.

## Author Contributions

**Hatice Toprak:** conceptualization, data curation, formal analysis, methodology, project administration, resources, writing–original draft, writing–review and editing. **Şükrü S. Toprak:** data curation, writing–review and editing.

## Ethics Statement

All procedures performed in studies involving human participants were in accordance with the ethical standards of the institutional and national research committee and with the Helsinki Declaration.

## Conflicts of Interest

The authors declare no conflicts of interest.
